# Prenatal Exposure to Mixtures of Nonpersistent Endocrine-Disrupting Chemicals and Angiogenic Biomarkers, Placental Function, and Fetal Growth

**DOI:** 10.1021/acs.est.5c13234

**Published:** 2026-03-09

**Authors:** Bethany Knox, Nuria Güil-Oumrait, Vishal Midya, Hana Vespalcová, Maria Dolores Gómez-Roig, Elisa Llurba, Sandra Márquez, Zoraida García Ruiz, Toni Galmes, Claire Philippat, Amrit Kaur Sakhi, Cathrine Thomsen, Jose Urquiza, Maria Julia Zanini, Payam Dadvand, Mireia Gascon, Ioar Rivas, Maria Foraster, Xavier Basagaña, Jordi Sunyer, Mariona Bustamante, Martine Vrijheid

**Affiliations:** † 310844 ISGlobal, C/Doctor Aiguader, 88, Barcelona 08003, Spain; ‡ Universitat Pompeu Fabra (UPF), C/Doctor Aiguader, 80, Barcelona 08003, Spain; § Spanish Consortium for Research on Epidemiology and Public Health (CIBERESP), 38176 Instituto de Salud Carlos III, C/Monforte de Lemos 3-5, Madrid 28029, Spain; ∥ Department of Environmental Medicine, 5925 Icahn School of Medicine at Mount Sinai, 1 Gustave L. Levy Place, Box 1091, New York, New York 10029, United States; ⊥ BCNatal. Barcelona Center for Maternal Fetal and Neonatal Medicine (Hospital Sant Joan de Déu and Hospital Clínic), University of Barcelona, 195999 Institut de Recerca Sant Joan de Déu, Passeig Sant Joan de Déu 2, Barcelona 08950, Spain; # Department of Food Safety, 25563 Norwegian Institute of Public Health (NIPH), PO Box 222 Skøyen, Oslo N-0213, Norway; ¶ Hospital de La Santa Creu i Sant Pau, Department of Obstetrics and Gynaecology, Institut D’Investigació Biomèdica Sant Pau - IIB Sant Pau, Sant Antoni Maria Claret, 167, Barcelona 08025, Spain; ∇ Primary Care Interventions to Prevent Maternal and Child Chronic Diseases of Perinatal and Developmental Origin Network (RICORS), RD21/0012/0003, Instituto de Salud Carlos III, Av. de Monforte de Lemos, 5, Fuencarral-El Pardo, Madrid 28029, Spain; ○ Spanish Network in Maternal, Neonatal, Child and Developmental Health Research (RICORS-SAMID, RD24/0013/0001), Instituto de Salud Carlos III, Madrid 28040, Spain; ⧫ Primary Care Interventions to Prevent Maternal and Child Chronic Diseases of Perinatal and Developmental Origin Network (RICORS), RD21/0012/0001, Instituto de Salud Carlos III, Av. de Monforte de Lemos, 5, Fuencarral-El Pardo, Madrid 28029, Spain; †† Unitat de Suport a La Recerca de La Catalunya Central, Fundació Institut Universitari per a La Recerca a L’Atenció Primària de Salut Jordi Gol i Gurina (IDIAPJGol), Gran Via de Les Corts Catalanes, 587, 08007 Barcelona, Spain; ‡‡ University Grenoble Alpes, Inserm U-1209, CNRS-UMR-5309, Environmental Epidemiology Applied to Reproduction and Respiratory Health Team, Institute for Advanced Biosciences, Site Santé, Allée des Alpes, La Tronche, Grenoble 38700, France; §§ Institute of Environmental Assessment and Water Research (IDAEA), CSIC, 18 26, Carrer de Jordi Girona, 18-26, Barcelona 08034, Spain; ∥∥ PHAGEX Research Group, Blanquerna School of Health Science, 16522 Universitat Ramon Llull, Barcelona, ES 08022, Spain

**Keywords:** chemical mixtures, fetal growth, placenta, Bayesian, environmental health

## Abstract

Exposure to endocrine-disrupting chemicals (EDCs) during pregnancy may influence the placenta and fetal growth; however, evidence is scarce regarding EDC mixtures, newer chemicals, and the role of angiogenic biomarkers and fetoplacental hemodynamics. We aimed to examine the associations between nonpersistent EDC mixtures and fetal growth, fetoplacental hemodynamics, and angiogenic biomarkers. We included 734 pregnant participants from the Barcelona Life Study Cohort (BiSC), Spain (2018–2021). Metabolites of phthalates, DINCH, insecticides, polycyclic aromatic hydrocarbons, pesticides, flame retardants, and parent compounds of phenols and parabens were measured in pools of week-long maternal urine samples at 18 and 34 weeks’ gestation. Penalized LASSO-type multigroup Bayesian Weighted Quantile Sum Regression estimated associations with fetal growth, fetoplacental hemodynamics, and angiogenic biomarkers. Birthweight z-score decreased with low-molecular-weight phthalate (LMWP) (β = −0.119; CrI −0.224, −0.008) mixtures and increased with organophosphate mixtures (β = 0.143; CrI 0.042, 0.245). LMWP exposure was also associated with altered hemodynamics and angiogenic biomarkers; angiogenic biomarkers mediated the relationship with birthweight z-score (ACME = −0.032; 95% CI −0.062, −0.009; *p* = 0.002). This comprehensive study suggests that mixtures of low-molecular-weight phthalates and organophosphate compounds may alter fetal growth and that angiogenic biomarkers may play a role as mediator.

## Graphical abstract

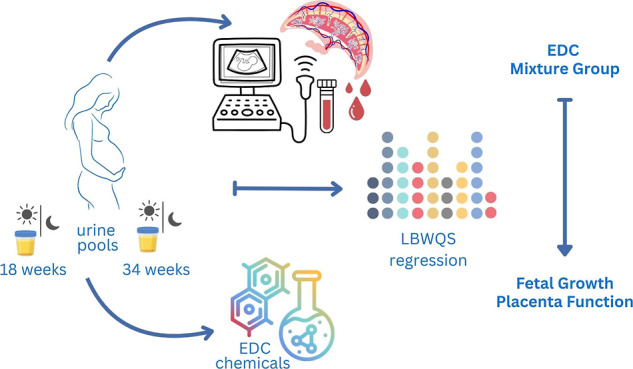

## Introduction

Endocrine-disrupting chemicals (EDCs) are widespread in the environment, leading to continuous human exposure. These compounds include nonpersistent (short half-life) chemicals such as phthalates, phenols (e.g., bisphenol A, triclosan), parabens, organophosphate (OP) pesticides and flame retardants, polycyclic aromatic hydrocarbons (PAHs), and emerging chemicals like bisphenol S (BPS) and 1,2-cyclohexane dicarboxylic acid diisononyl ester (DINCH).^1^ Exposure to nonpersistent EDCs occurs primarily through oral, respiratory, or dermal contact with common sources such as pesticide residue on food, drinking water, use of consumer and personal care products, and household dust.^2^ Of particular concern is exposure during pregnancy, a hormonally sensitive period during which EDCs may interfere with female endocrine function, thus impairing placental development and function, and disrupting fetal growth.^3^ As an essential endocrine organ, the placenta relies on precisely regulated proangiogenic (placental growth factor, PlGF) and antiangiogenic factors (soluble fms-like tyrosine kinase-1, sFlt-1) to ensure proper vascular development vital for fetal wellbeing.^3,4^ Epidemiological studies suggest that an increasing number of EDCs cross the placental barrier and are associated with adverse birth outcomes related to the placenta such as preterm birth, fetal growth restriction, and hypertensive disorders of pregnancy, among others.^3^ Low birthweight is associated with risk of disease later in life; however, disruptions to the maternal endocrine system during pregnancy may also alter programming of the fetal endocrine system, further influencing adult chronic disease risk.^5,6^ This raises critical concern about prenatal EDC exposure and underscores the need to investigate these exposures in pregnant persons and their offspring.

A limited number of studies have investigated associations between individual EDC exposures or chemical class, and repeated in utero measurements of fetal growth.^7,8^ Furthermore, despite evidence that pregnant individuals are exposed to complex mixtures of chemicals simultaneously,^9^ few have assessed the impact of EDC mixtures on in-utero fetal growth,^10–13^ and even fewer have incorporated chemicals from various chemical classes or examined placental function.^14,15^ An expanding body of experimental and epidemiological evidence demonstrates that EDCs likely influence multiple endocrine signaling pathways that underpin fetal–placental communication and programming, emphasizing the importance of examining endocrine organs such as the placenta.^16,17^ Yet, few studies have evaluated how nonpersistent EDCs affect fetoplacental hemodynamics or angiogenic biomarkers—key indicators of optimal fetal growth and placental function.^18,19^

Importantly, previous studies of nonpersistent EDCs and pregnancy outcomes mostly relied on one or a few spot-urine samples to assess EDC exposure, despite high intraindividual variability and short half-life. This approach may introduce considerable measurement error and exposure misclassification, leading to results biased toward the null. To more accurately characterize prenatal exposure, repeated samples are required.^20,21^

This study aimed to comprehensively examine the association between eight nonpersistent EDC mixtures (high- and low-molecular-weight phthalates, DINCH, insecticides, PAHs, OPs, phenols, and parabens) and angiogenic biomarkers, fetoplacental hemodynamics, and repeated, in utero fetal growth anthropometry. This was done by leveraging week-long pooled maternal urine samples at two time points during pregnancy for the measurement of exposure to EDCs.

## Methods

### Study Population

This study used data from the Barcelona Life Study Cohort (BiSC), in which 1080 pregnant participants were recruited during the first routine prenatal hospital visit (11–15 weeks of gestation) within the catchment area of three tertiary university hospitals in Barcelona, Spain, from 2018 to 2021. The recruitment procedure, follow-ups, and data collection methods are described elsewhere.^22^ Pregnant participants were included if they (i) had singleton pregnancy, (ii) were aged between 18 and 45 years, (iii) were able to communicate in Spanish/Catalan, (iv) were residents of the study area, and (v) planned to give birth in one of the recruiting hospitals. The current analysis was restricted to 734 pregnant participants with available data on EDC exposure for at least one time point and at least two fetal growth anthropometric and two uterine artery pulsatility index measurements (Figure S1). Participants provided written informed consent. Ethics approvals were obtained from the corresponding authorities in all the participating institutions and hospitals (Table S11).^22^

### Nonpersistent EDC Exposure Assessment

Data on chemical exposures was obtained at two time points during gestation (*n* = 695, median (IQR) weeks = 18.4 (4.1); *n* = 756, 34.7 (2.0)). Pregnant participants’ first and last voids of the day were collected for 6 consecutive days, and samples of equal volumes (1.8 mL) were pooled for each time point, approximately 18 and 34 weeks gestation, hereby averaging short-term variability in urine concentration. Urine samples were stored in 2 mL tubes at −80 °C until the time of analysis. Participants missing the collection of 2 or more samples over the 6 collection days were excluded. Chemical exposure assessment comprised bisphenols, parabens, OPs pesticides and flame-retardants, phthalates and nonphthalate plasticizers, and chlorpyrifos and pyrethroid insecticides. Selection was based on European biomonitoring studies and European Chemicals Agency (ECHA) candidate lists of chemicals of very high concern.^23^ A total of 44 compounds were measured as nanogram/mL in each urine pool (Table S1), and of these, 30 compounds had ≥50% of their measured concentrations above the limit of detection (LOD) and were included in this study (Tables S3 and S4). These 30 individual compounds formed 8 chemical mixtures (9 metabolites of high-molecular-weight phthalates [HMWPs], 3 metabolites of low-molecular-weight phthalates [LMWPs], 2 metabolites of 1,2-cyclohexane dicarboxylic acid diisononyl ester [DINCH], 2 metabolites of pyrethroid and chlorpyrifos insecticides, 4 metabolites of PAHs, 3 metabolites of OPs, and 4 phenols, and 3 parabens). Chemical analyses were performed at the Norwegian Institute of Public Health (Oslo, Norway) and used high-performance liquid chromatography and mass spectrometry (HPLC–MS–MS) for all chemicals, except phenols, PAHs, and pyrethroid and chlorpyrifos metabolites, which were measured using ultraperformance liquid chromatography–tandem mass spectrometry (UPLC–MS–MS) and ultraperformance liquid chromatography–quadrupole time-of-flight mass spectrometry (UPLC–QTOF). LODs spanned approximately 0.03–1.2 ng/mL. Quality assurance data, including LODs and lower and upper limits of quantification (LLOQ, ULOQ), are presented in Table S1. For individual chemicals with detection frequencies <50% below the LOD, we applied a single imputation when there was no signal detection from the HPLC–MS–MS/UPLC–MS–MS/UPLC–QTOF instrument using the fill_in function from the rexposome package in R (version 4.3.2).

### Fetal Growth, Fetoplacental Hemodynamics, and Angiogenic Biomarkers

Repeated estimated fetal weight (EFW) measurements were obtained via ultrasound using Hadlock’s formula III for prenatal visits at approximately 20, 32, and 37 weeks’ gestation, as previously described.^22^ Weight (grams) of the neonate measured by clinicians within 24 h of birth was obtained from the hospital medical record. Gestational age (GA) was calculated based on the crown-rump-length (CRL), measured using ultrasound examination at approximately 12th gestational week obstetric visit.^24^ To adjust for fetal size influenced by GA and fetal sex assigned at birth, z-scores were established using linear interpolation in reference to fetal growth charts established by the World Health Organization.^25^

Fetoplacental hemodynamics, assessed by pulsatility indices (PI) obtained via Doppler ultrasound, are commonly employed by gynecologists to evaluate placental function.^26^ Specifically, PIs of the uterine (UtA), umbilical (UmA), and middle cerebral arteries (MCA) reflect changes in perfusion to the fetoplacental unit and could be indicators of placental insufficiency, fetal compensatory adaptation, or intrauterine growth restriction (IUGR).^26–28^ Multiple PI parameters were obtained via Doppler velocimetry measurements conducted by a trained obstetrician. UtA PIs were measured at 20- and 32-week visits, and UmA and MCA measurements were obtained and used to calculate the cerebroplacental ratio (CPR) at the 32-week gestation visit.^22^ Z-scores based on GA were created for all PIs using published reference range values obtained from a Spanish population of uncomplicated pregnancies.^29^

Soluble fms-like tyrosine kinase (sFlt-1) and placental growth factor (PlGF) are antiangiogenic and proangiogenic proteins, respectively. An imbalance in these factors is indicative of placental insufficiency, and the sFlt-1/PlGF ratio is commonly used in clinical practice to detect preeclampsia, fetal growth restriction, or risk of preterm delivery.^30^ Peripheral blood from pregnant participants was obtained during the 32-week prenatal visit and centrifuged to obtain serum. Serum concentrations of sFlt-1 and PlGF were measured using automated electrochemiluminescence immunoassays on the Roche Cobas e601 platform (Roche Diagnostics GmbH, Mannheim, Germany). Reportable analytical ranges were 10–85,000 pg/mL for sFlt-1 and 3–10,000 pg/mL for PlGF. Limits of detection were 15 pg/mL for sFlt-1 and 10 pg/mL for PlGF. No measurements were <LOD. Intra- and inter-assay coefficients of variation were evaluated with PreciControl Multimarkers 1 and 2 (Roche Diagnostics, 2024) and were <5% in all assays. The ratio of sFlt-1 to PlGF was computed and then log_2_ transformed to achieve normal Gaussian distribution. Lastly, angiogenic biomarkers were standardized by GA using multiples of the median (MoM) and were calculated using quantile regression, in order to express how far an individual’s level deviates from the median of the same gestational age.^31^

### Maternal and Newborn Covariates

Maternal socio-demographic and pregnancy-related variables (parity, ethnicity, education, age, tobacco use) were collected through questionnaires administered by trained field technicians and medical record review during the first trimester at the routine 12th gestational week obstetric visit and at birth. Newborn sex assignment was collected from clinical records by BiSC clinicians.

### Statistical Analysis

Maternal EDC concentrations were log_2_ transformed. Pairwise Spearman’s correlation coefficients were calculated to examine the correlations between individual nonpersistent EDCs. Intraclass correlation coefficients (ICC) were calculated to measure the degree of consistency or variability of measurements within individual participants between the two exposure assessment periods (18 and 34 weeks’ gestation).^32^ To represent plausible influences graphically, directed acyclic graphs (DAG) based on a priori knowledge were used to select covariates (Figure S2a–c). Accordingly, all models were adjusted for the following: maternal BMI at 12-week gestation visit (kg/m^2^) as a proxy for prepregnancy BMI,^33^ maternal education completed (primary, secondary, university), self-reported ethnicity (European, Latin American, other), hospital of recruitment, maternal age at 12-week gestation visit (years), parity, and smoking during pregnancy (yes/no).^33^ To avoid the loss of study participants, we imputed missing values in covariate data by performing multiple imputations by chained equations (Table S2a). Twenty data sets were generated using the “mice” package in R, which assumes that data are missing at random.^34^

To estimate the association of EDC mixtures on fetal growth, fetoplacental hemodynamics, and angiogenic biomarkers, we used a penalized LASSO-type multigroup Bayesian Weighted Quantile Sum Regression (LBWQSR). LBWQSR is a novel extension of a Bayesian version of the Weighted Quantile Sum regression model that (1) considers the overall data-driven joint mixture effect and (2) allows concurrent inclusion of multiple groups of chemical compounds within the same mixture model while applying the elastic net-type penalties to prevent overfitting.^35,36^ Given the inter- and intragroup correlations between the chemicals, LBWQSR allows for the sharing of information between chemicals within a group, as well as between groups, through a shared variance term with a single origin. The model is implemented through RStan^37^ that uses the No-U-Turn sampler (NUTS) to obtain posterior distributions given a user-specified model and data.^38^ Mixtures were set to be ranked in quartiles. Further details regarding the characteristics and model convergence specifications are available in Table S2b.

We first applied LBWQSR models for each chemical mixture from the 18-week exposure assessment, along with each subsequent fetal growth, fetoplacental hemodynamic, and angiogenic marker follow-up time point (20, 32, and 37 weeks and at birth). We then repeated the LBWQSR models for each chemical mixture from the 34-week exposure assessment to examine the subsequent fetal growth and fetal hemodynamic outcomes (37 weeks and birth).

As a secondary analysis, we then incorporated repeated outcomes into a time-varying LBWQSR model to assess the overall effects across time points. The time-varying LBWQSR was used to associate 18-week exposure mixtures to fetal growth (at 20, 32, and 37 weeks, and at birth) and UtA z-score (at 20 and 32 weeks) and to associate 34-week exposure mixtures with fetal growth (from 37 weeks to birth). LBWQSR models were performed using the first of 20 imputed data sets with R version 4.4.1. (RStudio Team, 2023).^39^ Lastly, we conducted single-pollutant analyses using adjusted linear regression models for each single chemical exposure and birthweight z-score to support future risk assessment and to compare them with mixture-based results.

### Effect Modification Analysis

To explore whether fetal sex modified associations, we first stratified LBWQSR fetal growth models by sex at 20, 32, and 37 weeks gestation and birth to obtain effect estimates for each sex. Then, we calculated a mixture index for each group using the weights generated for each chemical within a mixture group from the LBWQSR stratified results and multiplied the weight by the individual’s concentration and summed these values to create the mixture index. The mixture index based on LBWQSR builds on Bayesian hierarchical LASSO-type models,^36,40^ implemented in Stan^41,42^ and on hierarchical Bayesian Weighted Quantile Sum regression formulations in which exposure weights follow a Dirichlet distribution.^43,44^ Because these approaches do not incorporate penalization of the mixture index and are vulnerable to multicollinearity, we integrate a hierarchical Bayesian WQS framework with LASSO-type shrinkage, as first implemented in an exposome data challenge.^45,46^ Finally, we regressed the exposure mixture index against the outcomes using linear regression models adjusted for the same covariates and included a multiplicative interaction term between the mixture index and the fetal sex. Statistical significance of effect modification was assessed using the *p*-value for the interaction term from these models.

### Mediation Analysis

We performed formal mediation analyses for the LMWP mixture measured at 18 weeks’ gestation for which we found significant associations with birthweight and fetoplacental hemodynamics to explore the potential mechanisms of action by the placenta using CPR and sFlt-1/PlGF measurements. We assessed the direct effect between the LMWP mixture index and birthweight by adjusting for CPR z-score, and separately, the sFlt-1/PlGF ratio. We then calculated the indirect effect by obtaining the product of coefficients between the effect of the exposure on the mediator and the mediator on the outcome using the mediation package in R.^47^ For each mediation analysis, we examined each of the relevant mediator-outcome in linear regression models and note that interpretation of mediation results relies on standard causal mediation assumptions as described previously.^48^ Statistical inference for the mediation effects was conducted using nonparametric bootstrapping with 10,000 resamples to estimate confidence intervals and *p*-values.

## Results

### Study Population Characteristics

Our study sample included 734 pregnant participants and their neonates from the BiSC cohort, described in Table 1. Compared to excluded participants, included participants were slightly more likely to be of European descent (76.3% vs 68.2%), to have a university degree (71.4% vs 64.5%), and to report taking supplements (omega-3 and folic acid). Included participants were on average 34.3 years of age at recruitment, nulliparous (59.7%), and nonsmokers (90.3%) (Table 1). Mean gestational age and weight at birth for neonates were 39.8 weeks and 3314.5 g, and occurrence of pregnancy pathologies (HDP, GD, or IUGR) was low (<5%). Of the EDCs included, MEP (18 weeks 40.4 ng/mL; 34 weeks 38.10 ng/mL) and MEPA (18 weeks 15.42 ng/mL; 34 weeks 17.85 ng/mL) had the highest median concentration. The majority of detection rates were above 80%, except BPS (59%), 9-PHOL + 1-PHOL(61%), and 1-OH-PYR (50%) (Figure 1, Tables S3 and S4). Mean concentrations and distributions were very similar between 18- and 34-week exposure time points (Figure 1).

**Table 1:** Study Population Characteristics in Comparison to the Subsample of Excluded BiSC Participants, Percent Missing of Covariates from the Included Population and after Imputation Averages^f^

	study population^a^ *N* = 734			excluded participants *N* = 346
	mean (SD) or *n* (%)	missing values *N* (%)	imputed data mean (sd) or *n* (%)	mean (sd) or *n* (%)
maternal characteristics				
age at delivery (years)	34.3 (4.6)	0 (0%)	34.33 (4.6)	33.65 (5.0)
ethnicity		0 (0%)		
european	560 (76.3%)		560 (76.3%)	236 (68.2%)
latin American	159 (21.7%)		159 (21.7%)	101 (29.2%)
other	15 (2.0%)		15 (2.0%)	9 (2.6%)
education completed		0 (0%)		
primary or less	30 (4.1%)		30 (4.1%)	19 (5.5%)
secondary	180 (24.5%)		180 (24.5%)	104 (30.1%)
university	524 (71.4%)		524 (71.4%)	223 (64.5%)
hospital		0 (0%)		
hospital A	9 (1.3%)		9 (1.23%)	82 (23.7%)
hospital B	278 (37.9%)		278 (37.9%)	153 (44.2%)
hospital C	447 (60.9%)		447 (60.9%)	111 (32.1%)
parity^b^		0 (0%)		
nulliparous	438 (59.7%)		438 (59.7%)	112 (56.6%)
1 child	240 (32.7%)		240 (32.7%)	59 (29.8%)
2 or more	56 (7.6%)		56 (7.6%)	27 (13.6%)
BMI^c^ (kg/m2)		47 (6.4%)		
underweight	16 (2.2%)		16 (2.2%)	4 (2.0%)
normal	439 (59.8%)		470 (64.0%)	108 (54.6%)
overweight	167 (22.8%)		180 (24.5%)	48 (24.2%)
obese	65 (8.9%)		68 (9.3%)	20 (10.1%)
pregnancy pathologies		48 (6.54%)		
HDP^d^	29 (4.0%)		30 (4.1%)	5 (2.5%)
	657 (89.5%)		704 (95.9%)	130 (65.7%)
gestational diabetes	31 (4.2%)		31 (4.2%)	10 (5.1%)
	655 (89.2%)		703 (95.8%)	125 (63.1%)
IUGR^e^	23 (3.1%)		27 (3.7%)	10 (5.1%)
	663 (90.3%)		707 (96.3%)	125 (63.1%)
smoking during pregnancy		17 (2.32%)		
none	663 (90.3%)		678 (92.4%)	139 (70.2%)
yes	54 (7.4%)		56 (7.6%)	16 (8.1%)
omega-3 supplement		367 (50%)		
no	293 (39.9%)		579 (78.9%)	33 (16.7%)
yes	74 (10.1%)		155 (21.1%)	7 (3.5%)
folic acid supplement		219 (29.84%)		
no	125 (17.0%)		173 (23.6%)	12 (6.1%)
yes	390 (53.1%)		561 (76.4%)	55 (27.8%)
GA sampling 2nd trimester	18.4 (4.1)	-	-	-
GA sampling 3rd trimester	34.7 (2.0)	-	-	-
offspring characteristics				
sex		0 (0%)		-
female	438 (49.7%)		372 (50.7%)	72 (36.4%)
male	444 (50.3%)		362 (49.3%)	79 (39.9%)
gestational age at visits (weeks)		0 (0%)		-
20 weeks	20.7 (0.7)	0 (0%)		20.7 (0.8)
32 weeks	31.7 (1.1)	0 (0%)		32.0 (1.2)
37 weeks	36.1 (1.2)	66 (9.0%)		36.2 (1.5)
at birth	39.8 (1.3)	0 (0%)		39.2 (2.2)
weight at birth (g)	3314.56 (457.0)	1 (0.1%)		3232.95 (626.3)
uterine artery (32w)	0.79 (0.2)	0 (0%)		0.73 (0.2)
cerebroplacental ratio (32w)	2.1 (0.5)	14 (1.9%)		2.1 (0.5)
sFlt-1/PlGF ratio (32w)	6.2 (14.8)	0 (0%)	-	-

a Study participants were required to have data for chemical exposures at either 18- or 34- weeks of gestation and at least 2 time points for fetal growth and uterine artery pulsatility index outcomes.

b Completed 20 weeks.

c BMI at 12 weeks.

d Hypertensive disorder during pregnancy.

e Intrauterine growth restriction.

f Totals may not add up due to missingness.

**Figure 1:**
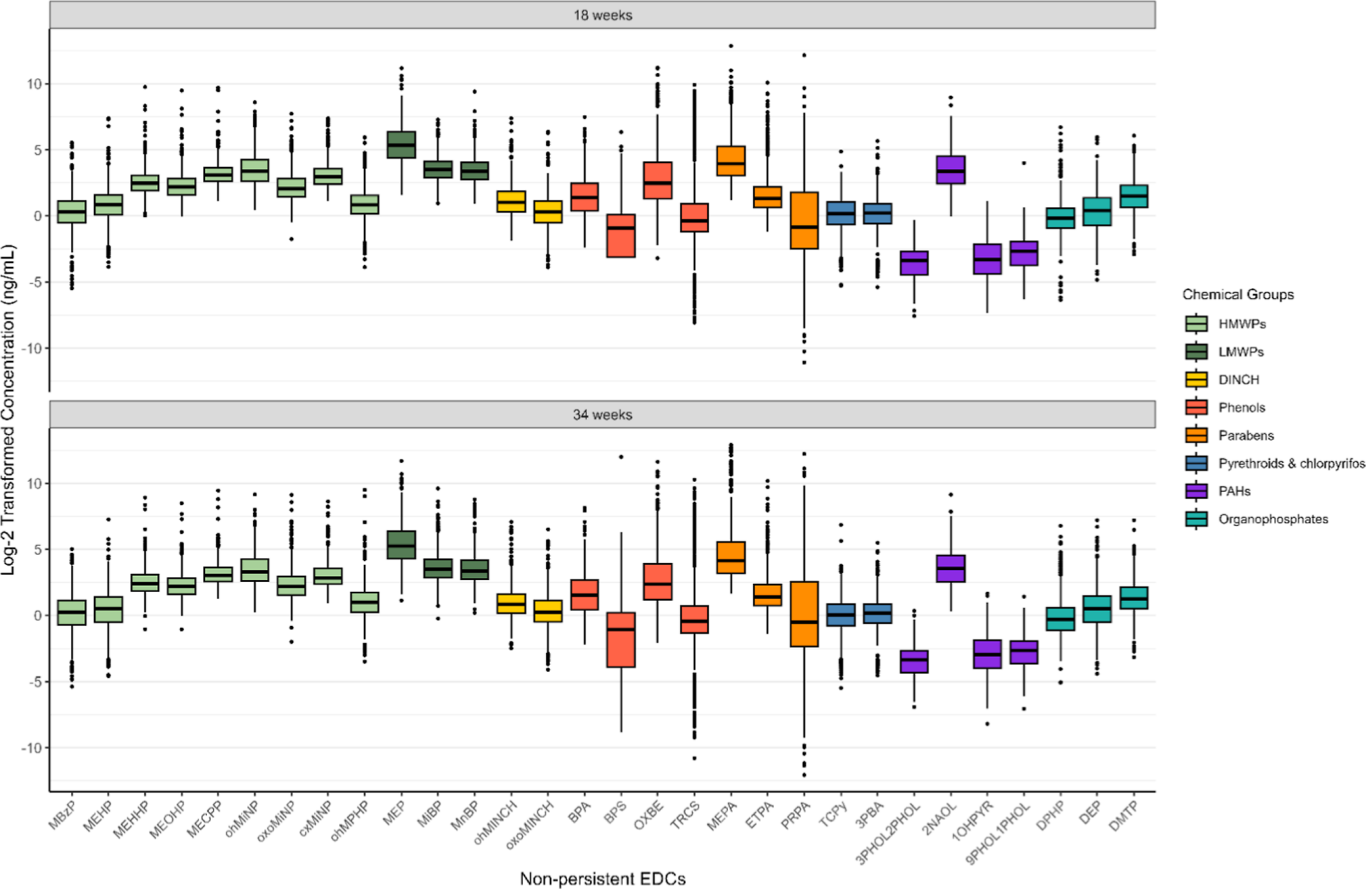
Log2-transformed non-persistent chemical exposure concentrations of the study population measured in pregnant participants’ pooled urine samples at 18- and 34-weeks’ gestation. All chemical abbreviations are defined in Table S1.

Correlations between the EDCs across chemical groups were mostly weak (*r* 0.0–0.5), however, between LMWPs and HMWPs, moderate to high correlations were observed (*r* ranging from 0.5 to 1.0) (Figure S3). Correlations between the two exposure time points (approximately 18 and 34 weeks) were low to moderate, with DEP and BPS showing the lowest (ICC = 0.12; 0.15), and OXBE and MBzP, the highest level of reproducibility over time (ICC = 0.64; 0.57). Reproducibility across pregnancy was generally low to moderate and comparable to previous studies, indicating within-person variability, as expected for rapidly metabolized chemicals and physiological changes across pregnancy.^32^

### EDC Mixtures and Fetal Growth

A quartile increase in exposure to the LMWP mixture at both 18- and 34-weeks’ gestation was associated with decreased birthweight z-score (β 18-week exposure = −0.119; credible interval [CrI], −0.224 to −0.008; β 34-week exposure = −0.116; CrI −0.217 to −0.016). MnBP (weight, 0.533) and MEP (weight 0.410) contributed most to the mixture at 18- and 34-week time points, respectively (Tables S5 and S6). We also observed a decrease in EFW z-scores at 32 and 37 weeks for the LMWP mixture, although not statistically significant (Figure 2, Tables S5 and S6). Similarly, a quartile increase in the phenol mixture at both 18- and 34-week exposure time points was associated with a decrease in EFW and birthweight z-scores across gestation (save for 20 weeks), but these estimates did not reach statistical significance (Figure 2, Tables S5 and S6). In contrast, we observed a statistically significant increase in EFW (37 weeks) and birthweight z-scores in response to a quartile increase in the OP mixture at 34 weeks (EFW: β = 0.141 [0.038–0.250]; birthweight: β = 0.143 [CrI 0.042–0.245]) (Figure 2). Weights were evenly distributed for EFW estimate, and DMTP (weight 0.451) contributed most to the mixture effect at birth (Table S6). The remaining mixtures (HMWPs, DINCH, parabens, pyrethroid, and chlorpyrifos, PAHs) did not show consistent associations with EFW or birthweight z-scores (Figure 2, Tables S5 and S6).

**Figure 2:**
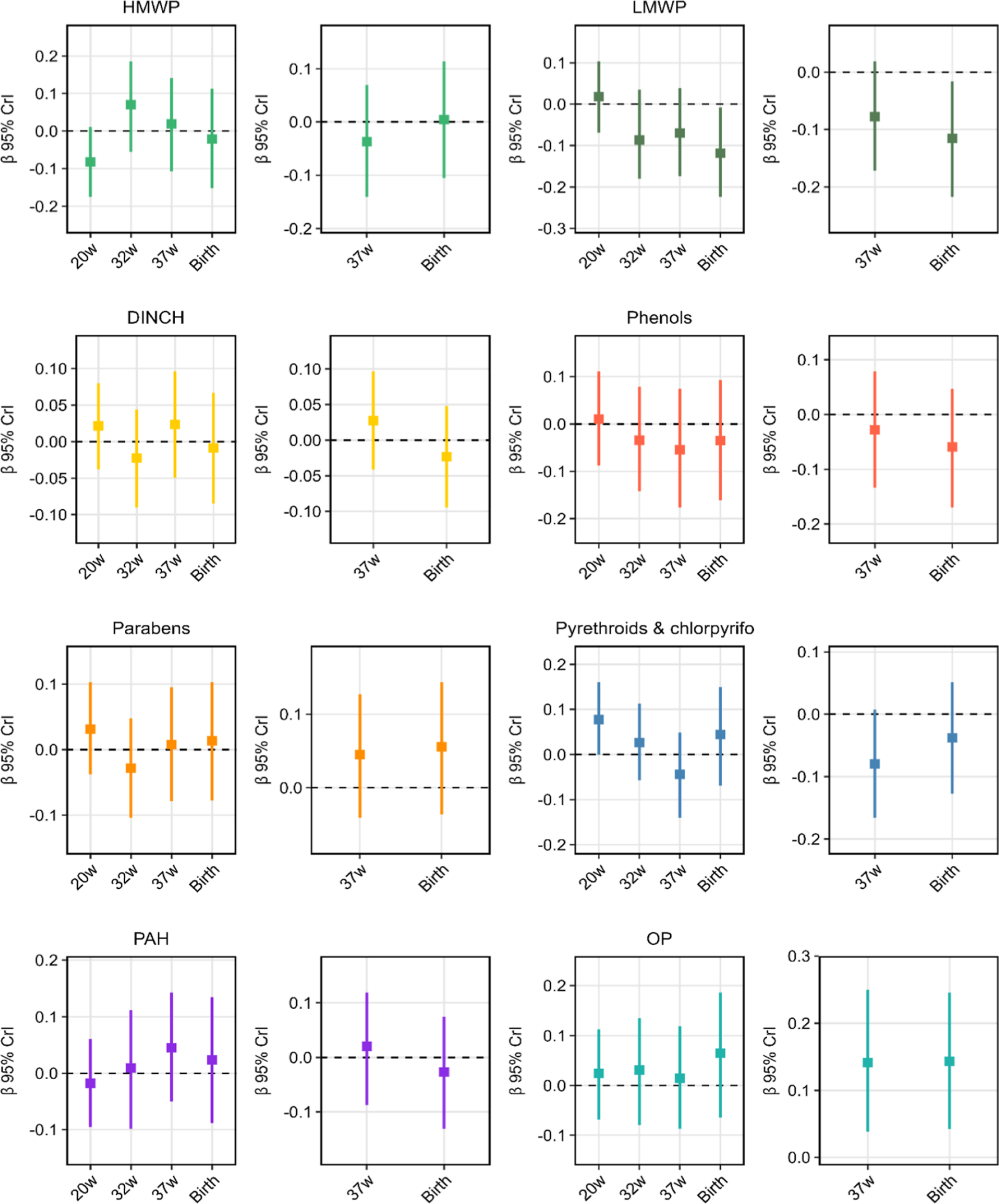
Joint effects of log2 transformed nonpersistent chemicals mixtures at 18 weeks of gestation (left) and 34 weeks of gestation (right) and estimated fetal weight z-scores at 20-, 32-, and 37-weeks’ gestation and weight at birth.

In the repeated outcome models, the estimates for associations of 18- or 34-week exposure with overall fetal growth did not reach statistical significance; however, patterns similar to the single-outcome time point analyses were observed (Table S7). For example, the 18- and 34-week LMWP and phenol mixtures were associated with a nonsignificant overall decrease in fetal growth z-score, and the OP mixture was associated with a nonsignificant overall increase in fetal growth z-score for the 34-week exposure time point (Table S7).

Stratifications by sex showed few differences between males and females and interaction tests were mostly nonsignificant (Table S8), with the exception of the 18-week phenol mixture and EFW and birthweight z-scores (*p*-interaction values <0.05, Table S8). For every 1-quartile increase in phenol mixture, birthweight z-score for male neonates decreased, while this was not the observed in female neonates (β males = −0.19; CrI −0.37 to −0.01, β females = 0.13; CrI −0.06 to 0.33, *p*-interaction <0.001). Single-pollutant analyses showed few statistically significant associations with birthweight z-score; however, significant findings were concordant with mixture model results. For example, MnBP exposure at 18 weeks (β = −0.10 CI −0.18, −0.02) and MEP exposures at 34 weeks (β = −0.05 CI −0.09, 0.00) were associated with a decrease in birthweight z-score, corresponding to the LBWQS models mixture contributions at each respective time point (MnBP 53%, MEP 41%).

### Nonpersistent EDC Mixtures and Placental Function

A quartile increase in the LMWP mixture at 18 weeks was associated with a decrease in CPR z-score (β = −0.184; CrI −0.322 to −0.037; MiBP weight 0.604), while the sFlt-1/PlGF ratio increased (β = 0.027; CrI 0.007–0.048; Table S9). UtA and UmA z-scores also increased with the LMWP mixture, though not significantly (Table 2). In contrast, the sFlt-1/PlGF ratio decreased in response to a quartile increase in the HMWP mixture (β = −0.028; CrI = −0.050 to −0.005) (Table 2). The remaining chemical mixtures showed no significant associations with UtA, UmA, or CPR z-scores or sFlt-1/PlGF ratio in response to 18-week exposures (Table S9). In the overall UtA model (UtA follow-up at 20- and 32-weeks’ gestation), associations with chemical mixtures varied in directionality, and none were significant (Table S7).

**Table 2:** Associations of Nonpersistent EDC Mixtures at 18 Weeks’ Gestation and Uterine Artery, Umbilical Artery, Cerebroplacental Ratio Pulsatility Indices and Angiogenic Biomarkers during Gestation^a^

	UtA 20 weeks		UtA 32 weeks		UmA 32 weeks		sFlt-1/PlGF 32 weeks		CPR 32 week	
mixture group	*n* = 604		*n* = 621		*n* = 630		*n* = 494		*n* = 616	
	β	CrI	β	CrI	β	CrI	β	CrI	β	CrI
HMWPs	–0.03	(−0.18, 0.12)	0.12	(−0.05, 0.29)	–0.02	(−0.09, 0.04)	–0.03	(−0.05, 0.00)	0.10	(−0.07, 0.27)
LMWPs	0.01	(−0.12, 0.14)	0.03	(−0.11, 0.18)	0.05	(−0.01, 0.11)	0.03	(0.01, 0.05)	–0.18	(−0.32, −0.04)
DINCH	0.04	(−0.05, 0.14)	–0.02	(−0.12, 0.08)	0.02	(−0.03, 0.06)	0.01	(0.00, 0.03)	–0.05	(−0.16, 0.05)
phenols	0.02	(−0.15, 0.19)	–0.01	(−0.20, 0.17)	0.00	(−0.08, 0.09)	0.01	(−0.02, 0.03)	0.01	(−0.18, 0.19)
parabens	0.02	(−0.11, 0.14)	0.06	(−0.08, 0.18)	–0.01	(−0.06, 0.04)	0.00	(−0.02, 0.02)	0.03	(−0.11, 0.17)
pyrethroids and chlorpyrifos	0.01	(−0.12, 0.15)	–0.01	(−0.14, 0.11)	–0.02	(−0.08, 0.04)	0.00	(−0.01, 0.02)	0.10	(−0.04, 0.24)
PAHs	–0.01	(−0.15, 0.12)	–0.10	(−0.25, 0.05)	–0.02	(−0.08, 0.04)	–0.01	(−0.03, 0.01)	0.05	(−0.10, 0.19)
organophosphates	0.04	(−0.14, 0.21)	0.05	(−0.10, 0.21)	0.01	(−0.06, 0.07)	0.01	(−0.01, 0.04)	–0.07	(−0.24, 0.09)

a Abbreviations: CrI, credible intervals.

### Mediation Analysis

Formal mediation analysis was performed to evaluate if the reduction in birthweight z-score observed in association with the LMWP mixture was mediated by CPR or the angiogenic biomarkers as well as if the reduction in CPR observed in association with the LMWP mixture was mediated by angiogenic biomarkers. We observed that the relationship between the LMWP mixture at 18 weeks and birthweight was significantly mediated via the sFlt-1/PlGF ratio (ACME = −0.032, 95% CI: −0.062, −0.009; *p* = 0.002) and that the proportion mediated was 38%. For the CPR z-score, we observed a small, nonsignificant indirect effect (ACME = −0.006, CI: −0.022 to 0.004; *p*-value = 0.276); the proportion mediated was 6% (Table S10). In a linear regression model examining the relationship between sFlt-1/PlGF ratio and birthweight z-score, we observed a decrease in birthweight z-score for every one-unit increase of the sFlt-1/PlGF ratio (β = −1.36 CI: −1.82 to −0.90, *p*-value <0.00), while for the CPR z-score, we observed a slight increase in birthweight z-score for every unit increase in the CPR (β = 0.10 CI: 0.02–4.20, *p*-value <0.00). When evaluating the mediation effects by sFlt-1/PlGF between the LMWP mixture and CPR, we found no mediation (ACME = 0.000, CI: −0.023 to 0.023; *p*-value = 0.958).

## Discussion

This study comprehensively examines the mixture effects of eight groups of nonpersistent EDCs on fetal growth, fetoplacental hemodynamics, and angiogenic biomarkers, using chemical mixture models and optimizing exposure assessment by relying on a large number of repeated urine samples during pregnancy. Median urinary concentrations in this birth cohort were generally within or below ranges reported in European human biomonitoring programs (e.g., HBM4 EU), which serve as a contextual benchmark.^1,49,50^ Exposure to a mixture of LMWPs (MEP, MiBP, MnBP), both at 18 and 34 weeks’ gestation, was associated with a decrease in birthweight. Conversely, exposure at 34 weeks to the OP mixture that included pesticides and flame retardants was associated with an increase in EFW and birthweight. With regard to placental function, the LMWP mixture was associated with a decrease in CPR and an increase in the sFlt-1/PlGF ratio, consistent with patterns of placental insufficiency. Lastly, our results suggest that the association between the LMWP mixture and birthweight is mediated by sFlt-1/PlGF angiogenic factors.

### HMWPs, LMWPs, and DINCH

In our study, exposure to a mixture of LMWP metabolites at 18- and 34-weeks’ gestation was associated with lower birthweight z-score, with MnBP and MEP contributing most to the mixture effects. These findings support existing moderate evidence linking prenatal exposure to both high- and low-molecular-weight phthalates—particularly MEP when assessed singularly—with decreased birthweight.^51,52^ However, inconsistencies across the literature are notable, especially in relation to sex-specific effects. A review by Kamai et al. identified five studies in which increased phthalate levels—of both high- and low-molecular-weight—were associated with increased birthweight, though only in male infants.^53^ Yet another meta-analysis reported that the association between MMP and lower birthweight was limited to female offspring.^54^ In our study, we found no evidence for sex specificity in the association between LMWP mixture and lower birthweight, but continued exploration of the role of fetal sex is important. Few studies assess phthalate mixtures in relation to repeated fetal growth ultrasound measurements during pregnancy and results vary.^8,11,12,15^ Santos et al. found that higher concentrations of LMWPs were associated with reduced fetal growth across gestation.^55^ A small (*n* = 254) US cohort found an overall reduction in fetal growth biometry in response to a quarter increase in phthalate mixture^12^ as did a Dutch cohort of comparable size to our Spanish cohort (*n* = 776).^15^ In contrast, Ouidir et al. found that a mixture of phthalates in a recent French cohort was significantly associated with an increase in estimated fetal weight during the third, but not the second, trimester.^11^ Fetal growth occurs unevenly throughout pregnancy, with early development focused on organs and skeletal structures, while the majority of adipose tissue accrues during the third trimester.^56^ This temporal variation may influence associations between chemical mixtures and fetal weight at different gestational stages and explain the inconsistencies between studies. Evidence regarding HMWPs, particularly DEHP, is also mixed.^53,54,57^ The review by Kamai et al. found that HMWP metabolites, especially MEHP and butyl benzyl phthalate metabolite (MBzP), were more often associated with altered fetal growth measurements across pregnancy rather than birthweight alone, though heterogeneity across study designs was high.^53^ In contrast, our study did not find associations between the HMWP mixture and fetal growth or birthweight (including sex-stratified analyses). Only two previous studies have assessed DINCH, a nonphthalate plasticizer replacement found in increasing levels since its introduction in 2002. One of the previous studies observed no relationship between fetal growth and DINCH exposure,^12^ similar to our study, while Ouidir et al. found an increase in metabolites of DINCH to be positively associated with fetal growth parameters during the second trimester.^11^ Inconsistencies across studies likely reflect differences in exposure timing and windows of susceptibility, outcome definitions, statistical models used, and exposure distributions across populations.

### Phenols and Parabens

While our study found no overall associations with the phenol or paraben mixtures, sex-stratified analyses revealed that increased phenol exposure was significantly associated with decreased fetal growth and birthweight in males only, with no effects observed in females. BPA has been widely studied, and while experimental evidence suggests it disturbs the normal growth and development of a fetus,^58^ epidemiological findings regarding other phenols and parabens and birth outcomes have been mixed and often depend on the outcome studied.^59–63^ Two systematic reviews concluded that most cohort studies examining BPA showed associations between higher maternal BPA concentrations and decreased indicators of fetal growth and adverse birth outcomes (e.g., low birthweight).^59,61^ However, a meta-analysis by Zhou et al. found that BPA was associated with increased birthweight.^63^ The systematic review by Guo et al. reported sex-specific associations between BPA exposure and size of neonates at birth.^59^ Importantly, BPA has been replaced by structurally similar compounds (i.e., BPS, BPF), which remain less studied.^58^ Our phenol mixture included well-studied phenols such as BPA and triclosan as well as the replacement compound BPS.

While parabens and phenols are structurally related, their exposure source and potentially endocrine effects may vary. Evidence regarding parabens is inconsistent with the context of fetal growth. A systematic review by Jamal et al. found that only one of 15 studies found a significant association between higher propyl paraben concentrations and decreased birthweight, only in male neonates.^60^ Differences may be seen across time points for health outcomes and singular paraben and phenolic compounds. For example, a large recent US birth cohort spanning 10 years found that prenatal exposure to phenols 2,4-DCP, TCS, and OXBE was associated with increased fetal growth during pregnancy but less so at birth.^64^ However, the same study found that methyl paraben was associated with decreased size at delivery but not with fetal growth during pregnancy.^64^

### Chlorpyrifos and Pyrethroids

We did not observe any consistent pattern of associations across single time points or in the overall models for chlorpyrifos and pyrethroid metabolites. These insecticides are used globally and existing epidemiological evidence has associated chlorpyrifos to adverse neurodevelopment, particularly in children.^65^ This led to its ban in the EU, and subsequent rise in pyrethroid use, which now accounts for approximately 80% of indoor insecticide use.^66^ Such widespread use prompted the prioritization of pyrethroids by HBM4 EU due to the limited knowledge on negative health effects in humans. Beyond neurotoxicity, chlorpyrifos and pyrethroids may have endocrine disruptive effects, as evidenced by reviews identifying interference with thyroid and adrenal gland function.^65,67^ While studies have examined prenatal exposure to these insecticides and birth size with mixed results, none, to our knowledge, have investigated repeated fetal growth measures.^66^

### Organophosphates

We found that an increase in the OP mixture exposure at 34 weeks gestation was associated with increased fetal growth at 37 weeks and with birthweight z-scores and that DEP and DMTP contributed most to the mixture. Although an increase in 18-week OP mixture was associated with increased fetal growth and birthweight z-scores, estimates were imprecise and did not reach statistical significance, suggesting limited detectability. OP concentrations in late pregnancy showed a higher central tendency and greater variability, which may increase the power to detect associations relevant to rapid fetal weight gain. The third trimester is a biologically sensitive window for fetal adipose tissue accumulation, making EFW at 37 weeks and birthweight more proximal outcomes for third-trimester exposure. In studies that examine OP pesticides, the results are mixed. The Generation R study measured the sum of mean concentrations across pregnancy of nonspecific dialkyl phosphates (DAP), metabolites of OP pesticides, and found that increased concentrations were associated with a decrease in EFW at 20 weeks, but not at birth.^13^ Similarly, a Danish cohort found no association between total DAP metabolites and birthweight.^68^ Our findings may reflect higher fruit and vegetable intake as OP exposure often results from pesticide residues on produce. One European study found that increased fruit consumption in mothers was associated with increased OP metabolites, while consumption of organic foods in children was associated with lower OP metabolites in the children.^69^ Our study did not include information related to organic food consumption. Furthermore, the presence of flame retardants in our OP mixture suggests environmental contamination beyond diet.^70^ Concerns about flame retardants are growing, given their wide use. A recent scoping review of eight studies examining OP exposure found no significant associations with birthweight, except for a small, nested case–control study in China,^71^ which reported a higher risk of low birthweight with higher OP exposure and a sum of multiple OP flame retardants.^72^ Another OPs study from a recent US case–control of 900 offspring from LIFECODES (2008–2018) observed positive associations between single chemicals and fetal growth parameters across pregnancy. However, associations with birthweight were null.^7^ These findings highlight the complexity of interpreting associations with OP exposure given its multiple sources and the mixed evidence across studies.

### PAHs

We found no associations between PAH metabolites (including phenanthrene) and fetal growth or birthweight z-scores. Exposure to PAHs and phenanthrenes is widespread due to numerous anthropogenic sources, including industrial processes and use of fungicides and insecticides.^73^ Phenanthrene, a prioritized substance under the HBM4 EU Action Plan and identified as 'of very high concern' by the ECHA, is one of many PAHs with limited human data, particularly regarding in utero fetal growth.^23,74^ For example, a recent systematic review found that across 31 observational studies included in the review, increased exposure to PAHs was associated with decreased birthweight. However, included studies used various exposure assessment methods (including dietary questionnaires, ambient air monitors, maternal biological samples urine, blood, placental and umbilical cord tissue), and no studies measured in utero fetal growth anthropometry.^75^ The same authors conducted a meta-analysis of 8 studies and found that an increase in 1-hydroxypyrene (1-OHP), principal metabolite of pyrene, was associated with a decrease in birthweight, though not significantly. Yang et al., in a meta-analysis, found no effect of ln unit μg/g (creatinine adjusted) increase in urine of detected 1-OHP and the risk of reduced birthweight.^76^ Continued research on PAHs, including phenanthrene, as well as their mixtures, is needed to clarify their potential impact on early development.

### Placental Hemodynamics and Angiogenic Biomarkers

The present study identified associations between LMWP exposure and biomarkers of placental dysfunction, including reduced CPR z-scores and increased sFlt-1/PlGF angiogenic factors, while the HMWP mixture was linked to a decrease in the sFlt-1/PlGF ratio. To our knowledge, this is the first study to evaluate comprehensive placental function using fetoplacental hemodynamics (UtA, UmA, and CPR PIs) and angiogenic biomarkers in relation to eight nonpersistent EDC mixtures. These findings provide new evidence that environmental exposures may impact critical pathways in placental development and function.

We observed a decrease in CPR z-score, consistent with the increase in the sFlt-1/PlGF ratio, in association with LMWP exposure. In the clinical setting, an abnormal CPR is typically defined as less than 1.08 or below the fifth percentile for gestational age of an established reference curve.^29^ An abnormal CPR reflects an adaptive mechanism in response to placental insufficiency, known as the “brain sparing effect”, wherein blood is preferentially shunted to the middle cerebral artery of the fetal brain.^77^ Results from the mediation analysis provide evidence of a mechanistic pathway, showing that the association between LMWPs and birthweight was partially mediated by the sFlt-1/PlGF ratio. Because these angiogenic proteins regulate vascular development, abnormal maternal serum levels in the third trimester can indicate fetal growth restriction related to placental insufficiency.^30^ Direct molecular evidence is limited; however, experimental studies indicate that phthalates induce inflammatory signaling and alterations to angiogenesis in trophoblasts, impairing proper placentation.^78^ Disruption to these regulatory proteins at the molecular level may help explain our observed associations as elevated sFlt-1/PlGF ratios are consistently linked to impaired placental angiogenesis and increased vascular resistance, reflecting downstream consequences for fetoplacental perfusion. The inverse association observed with the HMWP mixture suggests that different phthalate subclasses may affect distinct molecular pathways or regulatory feedback mechanisms in placental development. For example, LMW phthalates may preferentially disrupt endocrine and epigenetic regulation of trophoblast function, promoting antiangiogenic signaling and higher sFlt-1/PlGF ratios, whereas HMW phthalates may be more prone to effect maternal thyroid-related pathways that lead to increased pro-angiogenic responses, resulting in an inverse association with the ratio despite minimal effects on fetal growth.^79^ Collectively, these results suggest that phthalates may perturb angiogenic signaling pathways, potentially impairing placental perfusion during the third trimester.

Our findings align with prior research linking EDC exposure to other placental outcomes. For example, triclosan has been associated with placental weight,^13^ as has a sum of parabens, though only in male infants.^80^ Other studies have reported placental vascular resistance in the umbilical artery in negative association with MnBP and ΣDiNP, also limited to males.^14^ Disruption of metabolic hormones by EDCs may underlie these effects given the importance of endocrine signaling between maternal and fetal units and proper fetal growth.^81^ Further, our results regarding LMWPs and placental function support prior findings of associations between phthalate and phenol exposures and angiogenic biomarkers and placental vascular function, including altered PlGF and sFlt-1/PlGF ratios and impaired placental microvasculature.^18,19,82,83^ The current findings underscore the need for further investigation of placental function in relation to EDC exposure. Doppler ultrasound assessment of UtA, UmA, and MCA PIs and angiogenic biomarkers provides valuable insight into placentation and fetal well-being, potentially offering a more granular view of fetal growth.

### Strengths

The current study has several strengths. First, we had a comparably large sample size for the analysis of chemical mixtures and repeated in utero fetal anthropometry. Previous sample sizes from other similar studies were more modest (*n* = 254, Stevens et al. 2023; *n* = 478, Ouidir et al. 2024).^11,12^ Second, few studies have examined such a broad array of chemicals across multiple classes to address persisting uncertainties in health risks of emerging chemicals.^74^ Our study measured chemicals prioritized by both HBM4 EU and ECHA, contributing critical data on understudied substances, such as pyrethroids, phenanthrenes, and replacement compounds, such as DINCH (a phthalate alternative) and BPS (a BPA substitute). Third, our study greatly improved exposure assessment by collecting and pooling daily urine samples over 6 days in both the second and third trimesters. This approach significantly enhances measurement accuracy for nonpersistent, highly variable chemicals, reducing misclassification and improving reproducibility, as supported by prior research.^20,21^

Fourth, our study applied state-of-the-art mixture modeling to evaluate the combined effects of chemicals with potential synergistic effects, thereby enhancing real-world exposure characterization. A key strength of LBWQSR is its ability to estimate overall mixture effects in a data-driven manner without imposing directionality constraints while accounting for multicollinearity and providing interpretable weights that identify the most influential chemical contributors. Moreover, applying LASSO-WQS within the Bayesian framework incorporates prior knowledge to reduce overfitting, addresses multicollinearity and high dimensionality, and yields probabilistic effect estimates, enhancing interpretability in complex environmental exposure settings. Lastly, our study is among the first to utilize clinical placental function outcomes, recognizing the placenta as a critical endocrine organ that plays a vital role in fetal growth and is a likely target of EDCs. Our study facilitated the exploration of potential mediation pathways, advancing our understanding of how chemical exposures may impact fetal development.

### Limitations

Findings in the current study should be considered with some limitations. First, given the number of outcomes and tests performed, multiple testing adjustments may be warranted. However, within the Bayesian framework, multiple testing is inherently addressed by incorporating prior distributions that regularize parameters, thereby reducing the risk of false positives without the need for additional correction procedures. Second, the time-varying models in which we estimated the overall change in response to the chemical mixture and sex-stratified mixture models likely lacked sufficient sample size to produce reliable estimates and were therefore used as a secondary analysis. Additionally, exposure assessment began after the first trimester; thus, we could not evaluate early pregnancy or preconception exposures. A further limitation is that this study focused exclusively on perinatal health outcomes, although EDCs may have other adverse effects related to neurodevelopmental or metabolic dysfunction, which may be more sensitive indicators of toxicity than birth size. Lastly, we did not examine the combined effects of all chemical exposure groups, despite this being more representative of real-world exposure. Thus, it must be acknowledged that different chemical classes may affect human health via differing mechanisms and additive, synergistic, or antagonistic effects may occur simultaneously (e.g., LMWPs and OPs showed contrasting directions of associations).

## Supplementary Material



## Notes

Chemical analyte details including full names, abbreviations, limits of detection and quantification, and analytical methods for all measured nonpersistent EDCs; CONSORT diagram of the study population; Directed Acyclic Graphs for nonpersistent EDC exposures and fetal growth, fetoplacental hemodynamic, and angiogenic marker outcomes; imputation modeling and LBWQSR specifications; Spearman correlations and intraclass correlation coefficients of prenatal nonpersistent EDC urinary concentrations at 18 and 34 weeks gestation; distributions of angiogenic biomarkers, fetoplacental hemodynamics, and fetal growth outcomes; summary statistics of prenatal nonpersistent EDC urinary concentrations at 18 weeks and 34 weeks; joint mixture effects of prenatal nonpersistent chemicals at 18 weeks on estimated fetal weight and birth weight z-scores, at 34 weeks on estimated fetal weight and birth weight z-scores, at 18 weeks on overall fetal growth and uterine artery pulsatility indices, and at 18 weeks on uterine artery, umbilical artery, and cerebral placental ratio pulsatility indices and angiogenic markers; sex-stratified interaction analyses for EDC mixtures and fetal weight at 32 weeks, 37 weeks, and birth; mediation analyses of low-molecular-weight phthalate mixture at 18 weeks on birth weight and cerebral placental ratio; ethics committee approvals and informed consent details; and single-pollutant associations between prenatal nonpersistent EDC exposures at 18 and 34 weeks and birth weight (XLSX)

## Notes

Data sharing. Authors will share the data after the approval of the request by the BiSC steering committee and signing the data transfer agreement with the requesting institution. The request forms are available on the cohort Web site (https://projectebisc.org/en/home/). LBWQSR analysis code is available on GitHub: https://github.com/bknox19/Knox_2026.
